# New Zealand’s Integration-Based Policy for Driving Local Health System Improvement – Which Conditions Underpin More Successful Implementation?

**DOI:** 10.5334/ijic.5602

**Published:** 2021-04-23

**Authors:** Tim Tenbensel, Pushkar Raj Silwal, Lisa Walton, Reuben Olugbenga Ayeleke

**Affiliations:** 1 Public Policy, School of Population Health, Faculty of Medical and Health Sciences, University of Auckland, Bldg. 507, Level 3, 22–30 Park Ave, Grafton, Auckland 1023, New Zealand; 2 Health Systems Department, School of Population Health, Faculty of Medical and Health Sciences, University of Auckland, New Zealand; 3 Liggins Institute, Faculty of Medical and Health Sciences, University of Auckland, New Zealand

**Keywords:** New Zealand, inter-organisational collaboration, implementation, Qualitative Comparative Analysis, data sharing

## Abstract

**Introduction:**

The System Level Framework (SLMF) is a policy introduced by New Zealand’s Ministry of Health in 2016 with the aim of improving health outcomes by stimulating inter-organisational integration at the local level. We sought to understand which conditions that vary at the local level are most important in shaping successful implementation of this novel and internationally significant policy initiative relevant to integrated care.

**Strategy and Methods:**

We conducted 50 interviews with managers and clinicians who were directly involved in SLM implementation during 2018. Interview data was supplemented with the SLM Improvement Plans of all districts over the first three years of implementation. We used Qualitative Comparative Analysis (QCA) to identify the combinations and configurations of necessary and sufficient conditions of successful implementation.

**Results:**

We found that the strength of formal and informal organisational relationships at the local level were critical conditions for implementation success, and that while fidelity to the policy programme was necessary, it was not sufficient. Broader contextual features such as population size and complexity of the organisational environment were less important. The SLMF was able to deepen and widen inter-organisational collaboration where it already existed but could not mitigate the legacies of weaker relationships.

**Discussion:**

The two dimensions of implementation success, ‘Maturity of SLM Improvement Plan Processes’ and ‘Data Sophistication and Use’ were closely related. Broadly, our findings support the contention that integrated approaches to health system improvement at the local level require collaborative, trust-based approaches with an emphasis on iterative learning, including the willingness to share data between organisations.

**Conclusion:**

In the context of integrated care, our findings support the need to focus on establishing the conditions that build collaborative governance in addition to strengthening it when it already exists.

## Introduction

Two of the most important health policy trends of the twenty first century are the emphasis on integrated care and the focus on establishing policy frameworks for improving health outcomes. Although there are important connections between these developments, they have taken place largely independently of each other. The focus on health care integration has been on service design, organisational collaboration and alignment of funding mechanisms at the local and jurisdictional level. At around the same time, considerable interest in the measurement and monitoring of health outcomes has been driven by international organisations and researchers seeking to compare health system performance internationally. This also draws on concerted efforts to understand how changes in population health outcomes are attributable to health system activities [1]. Over recent years, this interest in health system improvement or performance is beginning to influence national health policy settings, particularly as it can be linked to efforts to reduce unwarranted variation in health outcomes.

While these developments have been largely separate, one of the key rationales for integrated care has always been the promise that improved integration will lead to improved health outcomes [2]. If it is reasonable to assess health systems in terms of health outcomes, (as well as efficiency and quality) then it is reasonable to expect integration between the parts of the health system to facilitate improvements in outcomes [3,4,5]. This broad proposition can be applied to multiple scales of analysis, ranging from the level of service delivery to the level of national health systems [6,7].

However, one of the challenges of research into integrated care has been to demonstrate the link between integration and improved health outcomes [8,9]. Health outcomes that are shaped by health systems and services, such as amenable mortality, vary within countries. If broad health policy and health system conditions are the same, but local health outcome indicators vary, it may be possible to identify features of local health system configuration that are linked to better or worse outcomes [10]. These analyses still need to factor in a range of socio-economic, demographic and geographic characteristics of local districts [11].

New Zealand has been at the forefront of developments to embed integrated care into health system performance frameworks at both the local [12,13] and national levels [14]. This has culminated in the 2016 introduction of the System Level Measures Framework (SLMF) which explicitly links integration and health system improvement [15]. The policy is novel inasmuch as it explicitly identifies health system integration and collaboration between organisations at the local level as a key driver of broader health system improvement. The introduction of this policy that emphasises the importance of local relationships and local approaches to health system improvement has provided the opportunity to investigate how local organisations take up the challenge of developing integrated approaches to improving health outcomes.

However, there are many potential barriers to inter-organisational collaboration including difficulties in negotiating shared accountability, and in access and availability of data to inform improvement [16]. Whether or not the organisations are willing to collaborate, share data and take a joint approach to service planning and delivery can be largely dependent on a range of local conditions including the configuration of organisations and the history of relationships between organisational leaders [17,18,19]. The introduction of the SLMF in New Zealand offers an opportunity to explore the implementation of a policy initiative that places inter-organisational relationships and collaboration at the heart of the approach to health system improvement.

### Background to the System Level Measures Framework

New Zealand’s health sector organisational environment presents both opportunities and challenges for the development of integrated approaches to health services and health system improvement. On the one hand, compared to many jurisdictions New Zealand’s policy and organisational frameworks provide significant opportunities for integration, and these opportunities have been exploited in some parts of the country [20]. On the other hand, the organisational landscape is highly fragmented, and unnecessarily over-complicated in some localities [21,22].

As of 2020, the key organisations in the New Zealand health sector were 20 decentralised government agencies known as District Health Boards (DHBs) which provide hospital services and purchase community-based health services including primary care. Between 70 and 75% of tax-funded health care passes through these DHBs [23]. The population catchments of DHBs range from 30,000 to over 600,000. Primary care services are predominantly provided by small business, for profit general practices. Almost all individual primary care practices are members of meso-level primary care organisations known as Primary Health Organisations (PHOs). DHBs have hospital and specialist service utilisation data, but primary care data is held by private primary care practices and PHOs. Integrating these sources of data has proven to be highly complex and challenging [24].

There has been a formal requirement since 2013 for organisations to form District Alliances at the local level. These have formally constituted Alliance Leadership Teams which is a governance arrangement consisting of senior leadership of DHBs, PHOs and, in some districts, other non-government health service providers. These District Alliances are often characterised by multiple workstreams across the organisations (Service Level Alliance Teams) that focus on substantive areas such as rural health or child health [25]. Building on the existence of District Alliances, the New Zealand Ministry of Health introduced the SLMF in 2016 [15]. The policy centres on six system-level measures defined nationally. They are measures that ‘require all parts of the health system to work together’, include a focus on children, youth and vulnerable populations, and are designed to connect to local, clinically led quality improvement initiatives [26]. Although the Ministry of Health has characterised the SLMF as an outcome-based approach, the six headline measures are a combination of outcome and process indicators.

**New Zealand’s System Level Measures (Ministry of Health 2016)**

- ambulatory Sensitive Hospitalisation (ASH) rates for 0–4-year olds (keeping children out of hospital) – *outcome*
- acute hospital bed days per capita (using health resources effectively) – *process*
- patient experience of care (person-centred care) – *process/outcome*
- amenable mortality rates (prevention and early detection) – *outcome*
- babies living in smoke free homes (a healthy start) – *outcome*
- youth access to and utilisation of youth appropriate health services (youth are healthy, safe and supported) – *process*

### Implementation of the SLMF

District Alliances have the responsibility for implementation of the SLMF [26,27]. Ministry of Health officials set out a process of developing annual SLM Improvement Plans based on co-operation and consultation between local organisations and stakeholders. The process for developing and deciding on contributory measures and actions was envisaged as broadly following quality improvement methodology in which participants use available health service utilisation data to identify the key drivers of headline measure performance at the local level, and then devise strategies for addressing them.

For example, one of the six headline indicators is ambulatory sensitive hospitalisation (ASH) for children aged 0–4. According to the policy logic, local health sector organisations, covering primary and secondary health care, and community-based services, examine their local data on ASH for 0-4-year-olds and identify the local drivers of the indicator. These could include respiratory infections, skin infections, dental caries and accidents. Local organisations are expected to collaboratively identify ‘contributory measures’ such as the rate of hospitalisations from respiratory infections. They then define actions aimed at reducing this rate, such as defining clearer clinical pathways, or developing pooled funding across providers for primary care home visits. New Zealand’s 20 District Alliances of DHBs and PHOs are required to develop local improvement plans and activities consisting of headline measure milestones, contributory measures, and defined actions [26,27].

Producing the SLM improvement plans requires a degree of co-operation and co-ordination across a range of local actors in District Alliances, which could include senior and middle level DHB managers, PHO managers, individual general practices, hospital clinicians, indigenous health service providers, pharmacists and midwives, and service providers focused on children and/or youth, with the range of relevant actors varying across different headline measures.

### Why might implementation vary?

In attempting to analyse and explain varying degrees of implementation success within jurisdictions, we draw upon a variety of disciplinary, theoretical and research approaches. Public policy implementation research, implementation science, and evaluation each emphasise the role of those with responsibility for implementation, and whether their motivations and behaviours are congruent with the broader policy logic [28,29,30]. This requires an understanding of the actions, motivations and sense-making of those directly involved in implementing the policy [28,31,32]. In any policy initiative that is implemented locally, it is likely that these processes of sense-making will be influenced by local factors which vary [33,34]. These factors include whether implementers see the initiative as fitting well with their aspirations and objectives, or whether they regard this policy as an additional imposition.

The role of context in implementation is central to understanding variation in implementation success [35,36,37,38]. Context can include proximate conditions that are likely to influence implementation, but which those directly involved in implementation have limited influence over. In researching any initiatives based on inter-organisational collaboration, contextual features are likely to be critical in shaping success [39]. Local inter-organisational climates vary in terms of their prior history [17]. In the New Zealand setting, a key dimension is whether relationships between DHBs and PHOs are built on transactional, principal-agent rules and routines, or on more relational practices [40]. The attitudes and practices of implementers of the SLMF are likely to be shaped by these histories, although it is possible that those involved in implementation could contribute to strengthening or weakening inter-organisational relationships.

Some contextual characteristics are well beyond the control of those involved in implementation [35,41] and as such it is useful to distinguish between inner contextual characteristics, and an outer context consisting of more distal elements. There are a wide range of political and social demographic factors that may influence SLMF implementation, including population characteristics, size and geography of a locality. In the case of the SLMF, smaller districts may struggle to develop the capacity to analyse data. Some conditions that promote and hinder effective collaboration are beyond the direct control of those attempting to develop networks [42]. In New Zealand there are 20 DHBs and 30 PHOs. DHB and PHO boundaries are not contiguous in many parts of the country, and PHOs sometimes compete with each other for primary care practice members [43]. Some DHBs interact with multiple PHOs, and some PHOs interact with multiple DHBs. As the funding of PHOs is determined by the enrolled population attached to member practices, some primary care practices ‘shop around’ for PHOs and this complicates the inter-organisational environment.

## Research Strategy and Methods

Our primary research objective was to understand how and why implementation success of the System Level Measures Framework varies across New Zealand’s districts.

### Research Objectives/Questions

Our specific research questions are:

1. To what extent does the implementation success of the SLMF vary between districts?
2. How can this variation in implementation be explained in terms of conditions that vary locally?
3. What are the implications of our findings for health system improvement approaches that are based on inter-organisational integration?

### Data collection

New Zealand’s health system structure provides the opportunity to research implementation across a larger, but manageable number of sites. making it possible to achieve a more comprehensive picture of variation. We aimed to collect data from all 20 health districts in New Zealand.

We developed a semi-structured interview schedule (Appendix 1) which was shaped by our prior understanding of the SLMF policy initiative and its relationship to other policy settings, policy documentation on the Ministry of Health website, and interview data from with government and non-government stakeholders involved in designing the SLMF, which was gathered during a preceding phase of the research.

Three districts adopted a combined approach to SLM implementation, resulting in a maximum of 18 possible cases. We conducted eight interviews from the districts with the combined approach, and 2-4 interviews from every other district, with the exception of one smaller district where organisational leaders declined to participate.

We conducted 50 interviews with managers and clinicians who were directly involved in SLM implementation during 2018, including 21 from DHBs, 27 from PHOs and two from other non-government organisations. Interviews were conducted in person, over the phone or over a video-link, and recordings were transcribed. Interview data was supplemented with the SLM Improvement Plans of all districts over the first three years of implementation [2016–17 to 2018–19]. Data from one district was incomplete, and was not included in the final analysis. This left us with data from 16 of the 18 implementation sites. Ethical approval was granted by the University of Auckland Human Participants Ethics Committee in 25 August 2017.

### Defining Implementation Success

**Figure 1:**
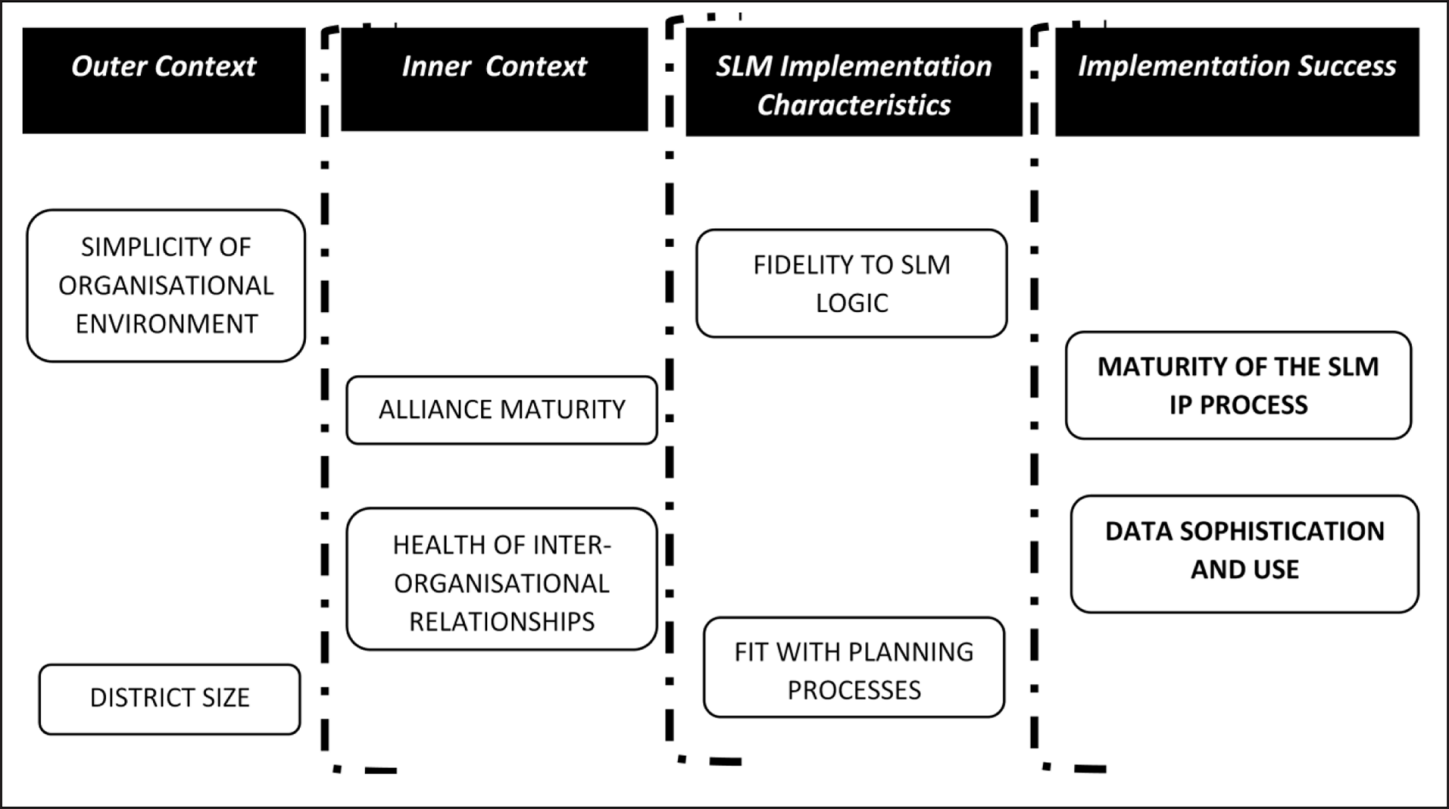
A framework for analysing the implementation success of SLMF.

Our conceptual model of implementation success is based on a combination of prior knowledge, and deductive and inductive coding of interview data. Interview transcripts were coded using NVivo 12 software. Criteria of implementation success needed to be sensitive to the phase of implementation, as our interviews were conducted between 20 and 28 months after the SLMF was introduced. Our conceptual model is depicted in ***Figure 1***. Appendix 2 provides full details of its development.

In the model we identify two dimensions of implementation success. The first pertains to implementation processes, specifically how organisational actors at the district level went about developing their SLM Improvement Plans (IPs). Our interview material and analysis of SLM IPs indicated that the degree of active engagement of primary, secondary, community and indigenous Māori health providers in the planning processes varied across districts. We labelled this condition as *‘Maturity of the SLM IP process’*.

The second criteria for evaluating successful implementation pertains to the management and use of data. Some districts reported relatively sophisticated data systems with good access to and use of central as well as locally generated data, they had high level of data sharing practices, and availability of data analytical capacity and capability within the alliance. These districts made more use of data in the process of setting the milestones and deciding on the contributory measures. We labelled this condition as *‘Data Sophistication and Use’*.

***Figure 1*** also depicts six elements that could possibly influence implementation success, and these are arranged in terms of their proximity to those directly involved in implementation.

- Two *SLM Implementation characteristics* are within the sphere of influence of those involved in implementation. *‘Fidelity to SLM Logic’* refers to the degree to which interviewees and SLM Improvement Plans reflected the focus on quality improvement, integration and reducing inequities. *‘Fit with Planning Processes’* indicated the degree to which SLM processes aligned with other DHB and PHO planning activities.
- Two *Inner Context* features which may be subject to some influence from implementers but are more significant as contextual factors shaping implementation. *‘Alliance Maturity’* denotes the formal aspects of inter-organisational relationships, while *‘Health of Inter-organisational Relationships’* covers the more informal features of these relationships.
- Two *Outer Context* features are not subject to any influence from implementers but represent potentially significant constraints. *‘District Size’* refers to the size of the district population, For the condition of *Simplicity of Inter-organisational Environment’*, where there was a single PHO relating to a single DHB, we regarded this structure as simple, whereas districts that had multiple PHOs and/or had PHOs that crossed district boundaries, had complicated structures.

### Data analysis

We used Qualitative Comparative Analysis (QCA) to analyse variation in implementation success in terms of the underlying DHB/PHO-specific local conditions. The QCA approach has been used elsewhere to examine implementation variation in public policy [44,45]. QCA also fits well with the tradition of realist evaluation of complex social phenomena [46] where relationships between causal mechanisms and outcomes are not fixed but are shaped by contextual factors [47,48].

The QCA approach requires the identification of one or more ‘outcome conditions’ which are influenced by configurations of ‘causal conditions’. These are the two conditions for implementation success outlined above ***Figure 1***. For datasets with 16 cases, QCA best practice was to limit the number of causal conditions to between four and six [49].

For each district (anonymised and then allocated a single letter identifier (e.g., District L) we allocated a score in the range of 1–4 for each outcome and causal condition (see Appendix 2). Then, we followed the standard 3-step process of fuzzy-sets QCA (fsQCA) as outlined by Charles Ragin [50]. Full details of the process of scoring and the application of QCA to our dataset are provided in Appendix 3.

## Results

### Outcome condition 1: Maturity of SLM Improvement Plan Processes

Mature processes entailed wider distribution of the workload and authority among the various groups and sub-groups involved in IP development where they had been allowed to select the contributory measures and decide on the milestones. These processes were either consistent across these dimensions or showed significant improvement in the early years of implementation. ***Table 1*** presents the results of fuzzy set QCA analysis for the successful implementation of SLM Improvement Planning processes.

**Table 1:** Configurations for Maturity of SLM Improvement Plan processes success (Outcome 1).


CONDITIONS	SOLUTIONS		

	1	2	3

DHB Size	•	⊗	•

Simplicity of I-O relation	⊗	•	-

Alliance maturity	–	•	•

Health of I-O relation	⊗	•	•

Fidelity to SLM logic	•	•	•

SLM fit	⊗	–	•

**Frequency of cases (District names)**	**2 (B, P)**	**6 (D, E, J, K, U, Z)**	**2 (R, Y)**

**Consistency**	**1.00**	**1.00**	**1.00**

**Raw coverage**	**0.31**	**0.59**	**0.39**

**Unique coverage**	**0.11**	**0.30**	**0.11**

**Solution coverage**	**0.83**		

**Solution consistency**	**1.00**		


**Notation:** (“•”) = presence of a condition; (“⊗⊗”) = absence of a condition; (“-”) = ‘don’t care’ situation where the condition is either present or absent [50].

Solutions 2 and 3 are comprised of a configuration that includes the two inner context variables (Alliance Maturity and Healthy Inter-organisational Relationships) within the district, combined with a high level of fidelity to the SLM logic (one of the two SLM implementation characteristics). Solutions 2 and 3 each represent a variant of this causal configuration. In Solution 2 (six cases), these conditions are supplemented by a simple inter-organisational context, but the fit with DHB and PHO planning processes does not matter. In Solution 3 (two cases), the inner context conditions combine with a large district size and fit with other DHB and PHO planning processes as a success pathway, but the complexity of the inter-organisational environment is less relevant.

Solution 1 which covers two cases (B and P), presents a very different pathway in which the inter-organisational relationship among the alliance members was less healthy and the perceived level of SLM fit with other planning processes is also low. Both cases were characterised by high fidelity to SLM logic, were relatively large in size and did not have simple inter-organisational environments. Overall, fidelity to the SLM logic is a condition that is shared across all solutions.

Our interview data provided further insight into these configurations. Having a well-functioning Alliance in terms of governance structures and allocated responsibilities within the Alliance supported the efforts of local implementers to engage a wider range of local stakeholders, and to develop a more distributed approach to SLM implementation. In District E, the link was articulated by DHB and PHO interviewees.

“So in establishing in particular the 2017–18 plan, the Alliance Leadership Team delegated a small sub-group of that which is the alliance management team, build that plan in the first instance” (District E, DHB).

“So at this stage in the true governance of the System Level Measures and the design, it’s DHB, PHO, general practice teams and [primary care practices] and [a Māori provider]. …And they are very much part of, are working with us as we design the contributory measures to achieve the System Level Measures” (District E, PHO)

Similarly, where inter-organisational relations were less robust, there was little scope for meaningful progress in SLM implementation.

“Yeah I have to say our Alliance Leadership Team hasn’t been very functional and for the last two years really has not been functional at all really. We’ve definitely met and System Level Measures was a part of that. But there was a lack of an integrated approach and a lack of a willingness to have all parties work together”, (District A, PHO).

### Outcome Condition 2: Data Sophistication and Use

Districts that scored highly on this criterion had developed relatively good quality data systems. Considering that we required a relatively even split in the numbers of successful and unsuccessful districts), the threshold for success on this dimension was the availability of shared data that was regarded as accurate, reliable, granular and timely. However, only a small subset of successful districts had progressed further in being able to use and analyse this data in a systemic way.

***Table 2*** presents the results of fuzzy set QCA analysis for implementation success measured in terms of the data sophistication and use.

**Table 2:** Configurations for achieving success: data sophistication and use (Outcome 2).


CONDITIONS	SOLUTIONS		

	1	2	3

DHB Size	⊗	•	•

Simplicity of I-O relation	•	–	⊗

Alliance maturity	•	•	⊗

Health of I-O relation	•	•	⊗

Fidelity to SLM logic	•	•	•

SLM fit	–	•	⊗

**Frequency of cases**	**6 (D, E, J, K, U, Z)**	**2 (R, Y)**	**1 (B)**

**Consistency**	**0.89**	**0.95**	**0.91**

**Raw coverage**	**0.61**	**0.45**	**0.27**

**Unique coverage**	**0.29**	**0.13**	**0.08**

**Solution coverage**	**0.85**		

**Solution consistency**	**0.92**		


**Notation:** (“•”) = presence of a condition; (“⊗”) = absence of a condition; (“-”) = ‘don’t care’ situation where the condition is either present or absent [50].

Nine of the ten districts that had mature SLM improvement plan processes also were regarded as successful on this criterion of data sophistication and use. As such, the success pathways for our second, substantive outcome are very similar to the pathways for relatively successful SLM Improvement plan processes.

Overall, there is no single necessary condition for this outcome even though the presence of the two inner context variables – a highly mature alliance and a high-trust/healthy inter-organisational field - were present in Solutions 1 and 2 in this analysis, covering eight of the nine successful cases. Solution 1 shows a common pathway to success for smaller districts, all of which had relatively simple inter-organisational contexts. Solution 2 covers larger districts in which SLM processes fit well with DHB and PHO planning, but inter-organisational context did not matter. Solution 3 shows an alternative pathway covering one case (District B) which was a large district with high fidelity to SLM logic, but all other conditions were absent.

From our interview data. the health of informal inter-organisational relationships appears to be crucial because it is a precondition of willingness to share data and make sense of it collectively. The clear link between inter-organisational relationships and data sophistication and use is articulated in the following extracts:

“Once contributory measures have been identified out, they were analysed for – the system side, technical side and patient side – then specific actions to influence them were identified. Very granular level of analyses had been conducted. The [alliance] has digitised data and the team looks at the [alliance] outcomes data every week” (District Y, DHB).

“So we have some PHOs who are very analytical savvy, and we’ve got one who’s a bit weaker. And for the one that’s actually the weaker, we’re doing a piece of work with them to strengthen their analytic ability. We otherwise have, for instance, invested in data visualisation tools as well… So, you know we can get more out of our information, so we do require them to do some of their own, but we also try to support them if there’s a gap” (District R, DHB).

Similarly, the link between poorer relationships and data use was clear in other districts.

“The DHB has still not been able to agree to a data sharing agreement with the PHO to access the raw data. …there’s no governance document for information sharing between primary and secondary. There’s a culture of distrust/fear/why the… … do you want to know about it?” (District L, DHB).

“I think what it is doing, it is shining a light on those areas where collaboration and sharing is not occurring, its forcing people to work their way through it… you know again it would not be unreasonable to say there’s been a significant amount of tension” (District P, PHO).

## Discussion

Overall, our most important finding is that successful implementation of New Zealand’s System Level Measures Framework is generally facilitated by well-functioning inter-organisational relationships (both formal and informal) and supported by fidelity to the logic of the SLM framework. Our results show that the two dimensions of implementation success were closely intertwined. In all but one district, the presence or absence of data sophistication and use went hand in hand with mature SLM processes.

This demonstrates the crucial importance of the willingness to share data between organisations. Only one district (District P) demonstrated successful implementation in one dimension (mature SLM IP process) and not the other (data sophistication and use). This suggests that it is possible, though rare, to have a collaborative planning processes without shared data.

### Factors within control of implementers

Implementers in most districts shared national policymakers’ assumptions about the centrality of integration, quality improvement and equity to the SLM framework. This fidelity to SLM logic was a necessary, but not sufficient, condition for successful implementation in terms of both process (SLM planning processes) and substance (data sophistication and use). While all relatively successful districts demonstrated fidelity to SLM logic, a handful of less successful districts also met this condition, and it was therefore not a sufficient condition for success. We found that the degree of fit with other formal planning processes at the district level was not a significant contributor to successful implementation, although it was absent for most districts that were less successful.

Taken together, the strong message is that those directly involved in implementing the SLM framework could not fully control implementation success by themselves. This may be because in all but the smallest districts, implementers were located in middle management rather than senior management of DHBs and PHOs. In some districts, the SLMF did promote collaborative relationships with colleagues across organisational boundaries developed for the specific purpose of developing SLM Improvement Plans. However, historical inter-organisational legacies could not be addressed at this level.

### Inner context

Inter-organisational relationship conditions had the largest impact on implementation success. With one major exception (District B), both the maturity of formal alliancing structures, and the more informal trust between DHBs and PHOs were present when implementation was successful, and absent when it was not. This also meant that fidelity to SLM logic only contributed to successful implementation when combined with relatively harmonious inter-organisational relationships.

We note that the two inter-organisational conditions correlated highly with each other. Only in district P were they different, with a mature District Alliance, but less healthy informal inter-organisational relationships. As noted above, District P was also the only district that was relatively successful in terms of SLM improvement plan processes, but less successful regarding data sophistication and use. This suggests that trusting relationships between organisations are needed to support data sharing between organisations.

The only major divergence from our findings about the crucial role of inter-organisational relationships was in District B, in which relatively successful implementation was achieved in the absence of mature alliances and healthy informal inter-organisational relationships. This district was markedly different from all other districts as those involved in implementation of the SLMF were not closely connected to the senior leadership of the Alliance and its constituent DHBs and PHOs.

### Outer context

Our analysis suggests that neither of the outer contextual features – namely the size of the district and the simplicity of the inter-organisational environment – were crucial elements of pathways to success. There were some pathways to relative success that were specific to smaller and larger districts respectively, and pathways to less success also had variants associated with both larger and smaller districts.

Most of the smaller districts that were relatively successful had simple inter-organisational environments. However, for larger districts, success on both criteria of implementation could also be achieved in more complicated settings. We should note, however, that the districts that were less successful and which had more complicated inter-organisational environments were clustered geographically, whereas the districts with complicated environments that were relatively successful were not part of this regional cluster.

We also found that district size could facilitate more sophisticated use of data in some circumstances and hinder it in others. Although many districts with smaller populations struggled to harness the analytical capacity to make sense of the data that was available, in many cases being smaller helped to facilitate other positive aspects of data interpretation and use that larger districts sometimes found challenging. These included sharing of utilisation data between primary care and DHBs.

### Limitations

We note some possible limitations to our research findings. Because we opted for breadth rather than depth of coverage, our interpretations of local conditions are mostly based on two or three interviews per district. It is possible that different interviewees would highlight different local features. This limitation is somewhat balanced by the fact that it was rare that interviewees within a district differed in their interpretations of the SLM implementation experience and the factors that affected it. Another consequence of choosing breadth over depth is that we are unable to unpack more specific aspects of collaborative practice that support implementation success.

Secondly, the local conditions and implementation experienced often changed significantly from year to year. Some districts reported that it wasn’t until the third cycle of improvement plans that key stakeholders in the district really began to understand the process and the potential of the SLM approach. It is possible that the situation in many districts (and the rating of conditions) may have been different if we had conducted our interviews significantly earlier or later.

Thirdly, the QCA approach also has inherent limitations, particularly in terms of the necessity of boiling down rich interview material and contextual information to a rating on a scale with a small number of points. This loss of information, however, is an inevitable trade-off for covering a greater number of cases, and that is why we drew upon interview data to support the QCA analysis.

## Conclusion

In broad terms, our findings support the contention that integrated approaches to health system improvement at the local level require collaborative, trust-based approaches with an emphasis on iterative learning [51,52]. Clearly, some districts were ready, willing and able to take up this challenge to build local, collaborative practices of health system improvement. These, however, were districts that had already been on a collaborative journey for some time, and with the introduction of SLMs were able to incorporate health system improvement with a focus on quality, integration and equity into their repertoire of collaboration.

But it was also clear that many districts did not possess the requisite conditions for this to happen, particularly when their local environment had been characterised by low-trust, conflictual relationships, either between competing PHOs, between DHBs and PHOs, or both. In many districts with weaker inter-organisational relationships, the SLMF brought people around the table and facilitated more collaborative working at amongst middle management and clinicians. While many respondents reported that inter-organisational collaboration improved over time in implementing the SLMF, the forging of relationships at the middle level did not jump-start more collaborative inter-organisational relationships at the senior management level where these had been more conflictual.

The important implication of this finding for integrated care is that a policy stimulus such as the SLMF has the potential to amplify the development of inter-organisational integration at the local level. The challenge of developing systems of health performance feedback was welcomed in districts that had a history of collaborative working. However, this policy stimulus did not appear to overcome negative inter-organisational dynamics where they were present.

The SLMF is an example of a policy approach which provides broad guidelines but places the onus on local implementers to make it work. In such cases, implementation success is strongly connected to pre-existing local conditions [34,53,54]. If the pre-existing state of inter-organisational relationships is the key to successful implementation, it is unlikely that successful implementation practices will scale and spread spontaneously. The inter-organisational context is often deeply influenced by history, clashes of organisational culture, and ingrained relationships between key players.

In the context of integrated care, our findings support the need to focus on the conditions that *build* collaborative governance [16,55] in addition to strengthening it when it already exists. While we are not able to judge whether the SLMF will ultimately generate a collaborative and integrated data-driven feedback loop of health outcome measurement, monitoring and decision-making, our research has shown that positive inter-organisational relationships are a pre-requisite for making any progress towards this goal.

## Additional Files

The additional files for this article can be found as follows:

10.5334/ijic.5602.s1 Appendix 1. Interview Schedule for District Participants.

10.5334/ijic.5602.s2 Appendix 2. Development of Conceptual Framework.

10.5334/ijic.5602.s3 Appendix 3. QCA analysis, technical appendix.
