# Supplementary material for: New Zealand’s Integration-Based Policy for Driving Local Health System Improvement – Which Conditions Underpin More Successful Implementation?
Source: Int J Integr Care. 2021 Apr 23;21(2):8. doi: 10.5334/ijic.5602 (PMC8064288; doi:10.5334/ijic.5602)
Supplement: Appendix 1. — Interview Schedule for District Participants. [file ijic-21-2-5602-s1.pdf]

Appendix 1

Interview Schedule for District Participants

1) Please tell me about your current/past position(s) and your role/involvement in the implementation of the System Level Measures framework in your district
2) How have District Alliances between DHBs, PHOs and other organisations developed in your district over the past 5-8 years?
3) In your view, what is the System Level Measures framework meant to achieve, and how is it meant to achieve it?
4) Describe/comment on your experience in developing SLM Improvement Plans for your district in 2016 and 2017.
5) How does the SLM approach fit (or not fit) with existing approaches to measuring and monitoring performance in your district?
6) Comment on the capacity of organisations in the District Alliance to collaborate in the development of SLM Improvement Plans, and the implementation of those plans
7) Comment on the capacity in your district for organisations (individually and collectively) to gather and interpret relevant health outcome and health services data in order to:
    - Establish SLM benchmarks for the headline indicators
    - Choose contributory measures
    - Determine District Alliance funding priorities
    - Make decisions about services (further investment, disinvestment)
    - Attribute changes in SLM indicators to initiatives at the district level
8) What improvements/developments to the SLM framework would you like to see?
    - Probe what alternatives to the SLMs that interviewees might suggest? If so, how would these work for a District Alliance?
