# Supplementary material for: New Zealand’s Integration-Based Policy for Driving Local Health System Improvement – Which Conditions Underpin More Successful Implementation?
Source: Int J Integr Care. 2021 Apr 23;21(2):8. doi: 10.5334/ijic.5602 (PMC8064288; doi:10.5334/ijic.5602)
Supplement: Appendix 2. — Development of Conceptual Framework. [file ijic-21-2-5602-s2.pdf]

Appendix 2: Development of Conceptual Framework

In our first step of organising the interview and document data we created parent nodes in nVivo based on each of the questions from the interview schedule. Child nodes were created within each parent node using a combination of deductive and inductive strategies.

For example, when asking respondents for their understanding of the logic of the SLMF, we were specifically looking for mentions of quality improvement, integration and a focus on reducing inequities between population groups, as these were important objectives of the policy as gleaned from documents and interviews. For other codes such as how local data was collated, shared, used and interpreted, an inductive strategy was more appropriate. Initially, two people coded six transcripts, and then codes were iteratively refined. Once the structure of parent and child nodes were agreed by the team, one researcher (PS) coded all transcripts. Another team member (RA) then coded all SLM Improvement plans using the same coding structure A detailed map of the final coding structure is provided in Table A2.1.

Two of the parent node categories, based on responses to interview questions 4, 6 and 7, provided the bases for indicators of successful implementation. The first pertains to implementation processes, specifically how organisational actors at the district level went about developing their SLM Improvement Plans. Our interview material and analysis of SLM IPs indicated that the degree active engagement of primary, secondary, community and indigenous Māori health providers in the planning processes varied across districts. We labelled this condition as *'Maturity of the SLM IP process'*.

The second criteria for evaluating successful implementation pertains to the management and use of data. Some districts reported relatively sophisticated data systems with good access to and use of central as well as locally generated data, they had high level of data sharing practices, and availability of data analytical capacity and capability within the alliance. These districts made more use of data in the process of setting the milestones and deciding on the contributory measures. We labelled this condition as *'Data Sophistication and Use'*.

We then looked to our data for features at the local level that could possibly influence these dimensions of successful implementation. Four other parent codes were selected, which we categorised into two sets. First, aspects of implementation that were directly related to the sense-making, actions and behaviours of those directly involved in implementation, labelled as '*Fidelity to SLM logic*', and perceptions of the implementers on how well the SLMF fit with other health sector policies and requirements, particularly around service planning in DHBs and PHOs, labelled as '*SLM fit with planning processes*'.

The second set pertained to inter-organisational relationships at the local level. One parent code contained information about the nature of District Alliances, labelled as *'Alliance Maturity'*, and another parent code containing information about the informal relationships between health sector organisations in the district, labelled as *'Health of inter-organisational relationships'*.

The final components of our conceptual model of implementation success were drawn from other sources. As outlined in the earlier discussion, we identified two variable features of districts that could shape implementation success. The first was the size of the district's population, and the second was the broad structure of the inter-organisational environment. Where there was a single

PHO relating to a single DHB, we regarded this structure as simple, whereas districts that had multiple PHOs and/or had PHOs that crossed district boundaries, had complicated structures.

Table A2.1 : Data organisation, coding, and building a conceptual model of implementation

**First order categories**			**Second order categories (Success and Causal Conditions)**	**Model of Implementation (Implementation factors)**
**Key themes identified**	**Description of the themes**	**Data sources**		
Simplicity of the Inter Organizational relationship	• Number of PHOs and DHBs in the alliance structure	Ministry of Health website	**Simplicity of the inter-organizational environment**	**Outer Context**
District size*	• (DHB) population<br>o Category 1: > 400,000,<br>o Category 2: 200,000 – 400,000,<br>o Category 3: 100,000 – 200,000, and<br>o Category 4: < 100,000	Ministry of Health website	**District size**	
Health of I-O Relationship	• How is the overall relationship of the alliance members?<br>o ‘Single plan’ to ‘No trust’ – feeling<br>o DHB vs. PHO transactional relationship,<br>o Good working relationships between DHBs, PHOs, other providers,<br>o Level of trust between organisations, and between key personnel	Interview data and SLM Improvement Plans (2016/17 – 2018/19)	**Health of inter-organisational relationships**	**Inner Context**
Maturity of the Alliance	• Whether a well-structured and functional alliance exists at the district or not?<br>• Potential variation dimensions - ALT structure, History, Alliance complexities and SLAT by broad issues or by SLM headings	Interview data and SLM Improvement Plans (2016/17 – 2018/19)	**Alliance Maturity**	
Alignment with DHB planning processes	• Perception of the respondents on whether SLM fits in broader DHB plans and processes or not	Interview data and SLM Improvement Plans (2016/17 – 2018/19)	**SLM fit** with planning processes	**Implementation Characteristics**
Alignment with PHO planning processes	• Perception of the respondents on whether SLM fits in broader PHO plans and processes or not	Interview data and SLM Improvement Plans (2016/17 – 2018/19)		
Emphasis on Equity	• Understanding and perception of the respondents on focus of the SLM on Equity:<br>o Whether SLMF is for equity or	Interview data and SLM Improvement Plans	**Fidelity to SLM logic**	

	not (MOH side)?<br>o Whether SLMF is for equity or not (District alliance side)?	(2016/17 – 2018/19)		
Emphasis on Integration	• Understanding and perception of the respondents on focus of the SLM on Integration (integrated delivery of services (primary, secondary and community services):<br>o Whether SLMF is for Integration (integrated delivery of services (primary, secondary and community services) or not (MOH side)?<br>o Whether SLMF is for Integration (integrated delivery of services (primary, secondary and community services) or not (District alliance side)?	Interview data and SLM Improvement Plans (2016/17 – 2018/19)		
Emphasis on Quality Improvement	• Understanding and perception of the respondents on focus of the SLM on Quality Improvement:<br>o Whether SLMF is for QI or not (MOH side)?<br>o Whether SLMF is for QI or not (District alliance side)?	Interview data and SLM Improvement Plans (2016/17 – 2018/19)		
Inclusiveness of SLM Improvement plan	• Who are officially included in the alliance structure (DHB managers, PHO managers, DHB clinicians, PHO clinicians, Midwives, Pharmacy, others?<br>• Whether the alliance members have active participation in the SLMF planning process or not? e.g. Inclusion of the alliance members in the SLMF improvement planning processes	Interview data and SLM Improvement Plans (2016/17 – 2018/19)	**Maturity of SLM Improvement processes**	**Implementation Outcomes**
Design of SLM Planning approaches	• How were the SLMF plans developed (the last two or three plans): through the working groups meetings and/or one-off workshops?	Interview data and SLM Improvement Plans (2016/17 – 2018/19)		
Distribution of workload and authority across organisations	• Whether there were specific SLM working/groups or not?<br>• What is the sign-off/approval process?<br>• Whether there are existence of collaborative sub-groups, leadership, and collaboration deepening over time or not?	Interview data and SLM Improvement Plans (2016/17 – 2018/19)		
Data	• Whether a good quality (Accuracy,	Interview	**Data**	

availability	Reliability, Granularity, Timeliness) data is available or not?<br>• Whether data generated at different levels/organizations are well linked or not (compatible, privacy and trust ensured, technically sound)?	data and SLM Improvement Plans (2016/17 – 2018/19)	**Sophistication and Use**	
Sense-making of data	• Whether the alliance has analytical capacity in terms of resources and expertise or not?<br>• Whether data has been used or not for planning purposes (identifying patients, identifying conditions, deciding priorities, defining actions, identifying inequities etc.)<br>• Whether there were clear feedback and monitoring mechanisms or not (attribution, linking actions to CMs, Linking CMs to headlines, and tracking improvement)?	Interview data and SLM Improvement Plans (2016/17 – 2018/19)		

We developed a framework matrix for each district in which interview data was summarised against each 'child node' category. We then allocated a rating from 1 (low) – 4 (high) in relation to each child node concept for each interviewee, then produced an overall rating for each district by aggregating individual ratings in each district.

We then aggregated the child node ratings into a summary score (1 – 4) for each condition (parent node). For example, for 'fidelity to SLM logic' the component scores for 'equity', 'integration' and 'quality improvement' were consolidated into a single score on the four-point scale for each district. Each condition rating was arrived at by two members of the research team (independently first and then agreed together). Any remaining disagreements on the scores were discussed in the wider team, revisited the scoring criteria/supporting information, and resolved. These ratings were then fed back to our research participants after which we made one further change.

For the two outer context condition variables, a high score on population size indicates a larger population (> 400,000), and the highest score for 'simplicity of organisational environment' indicates a single PHO per DHB.

Table A2.2: Final scores for each district

District codes	Outer Context		Inner Context		Implementation Characteristics		Implementation Success	
	District size	Simplicity of inter-org. environment	Alliance Maturity	Health of inter-org relationship	Fidelity to SLM logic	SLM Fit with Planning	Maturity of SLM Improvement Planning	Data sophistication and use
A	1	3	1	1	2	3	*2*	*2*

B	4	1	2	2	4	2	***4***	***4***
D	1	4	3	4	3	4	***4***	***3***
E	2	4	4	4	3	4	***4***	***3***
G	4	1	2	1	2	1	***2***	***2***
J	2	3	3	4	3	2	***3***	***3***
K	2	3	3	3	3	1	***3***	***2***
L	3	3	2	2	2	2	***2***	***1***
P	3	2	4	2	4	2	***3***	***2***
Q	2	2	2	2	3	2	***3***	***2***
R	3	2	4	4	4	4	***4***	***3***
S	1	3	1	2	3	2	***1***	***2***
U	2	3	4	4	4	3	***3***	***3***
X	2	2	2	2	3	2	***2***	***3***
Y	4	3	4	4	3	4	***4***	***4***
Z	2	4	3	3	3	2	***3***	***3***
