# Supplementary material for: New Zealand’s Integration-Based Policy for Driving Local Health System Improvement – Which Conditions Underpin More Successful Implementation?
Source: Int J Integr Care. 2021 Apr 23;21(2):8. doi: 10.5334/ijic.5602 (PMC8064288; doi:10.5334/ijic.5602)
Supplement: Appendix 3. — QCA analysis, technical appendix. [file ijic-21-2-5602-s3.pdf]

Appendix 3 – QCA analysis, technical appendix

QCA was designed by Charles Ragin (1), as a tool for explaining patterns of similarity and differences that can be used to analyse and explain variation between a small to medium number of cases. It is a useful approach to study causality in complex policy issues that deal with 'cases', seeks to gather greater insights into the contextual information about the cases thereby capturing it's complexities through formal and systematic case comparison(2). Ragin (3) argues that analyses that take a holistic perspective should concentrate on 'cases' rather than the 'variables'. It is the cases that are actually the 'actors' even though the various 'conditions' (variables) relevant to cases interact to produce results.

QCA analysis is based on set-theoretic relations among the 'cases' and the 'conditions' where various possible configurations of the conditions are studied in order to identify 'necessary' and 'sufficient' conditions. Rather than aiming to identify a single, 'best-fit' explanation based on linear relationships between independent variables, QCA explores the possibility of multiple causal pathways. Another important feature of QCA is that it allows for causal asymmetry. This means that the explanations (causal pathways) for implementation failure need not be the mirror-image of the explanations of success.

We followed the standard 3-step process of fuzzy-sets QCA (fsQCA) as developed by Charles Ragin (4).

Step 1: Calibration (transforming variables into sets):
The aggregated scores of the variables were transformed into scores based on the membership values of 0 and 1 respectively using direct calibration method by using fsQCA software(5). The upper limit was set at fuzzy score = 0.95 (threshold for full membership in the target set) = 4 in our case, the lower point at fuzzy score = 0.05 (threshold for non-membership in the target set) = 1 in our case and the cross-over point in the target set at fuzzy score = 0.5 = 2.5 in our case. We did not follow any sample-based calibration which is generally discouraged(6, 7). This method transforms the scores based on the log odds of full membership(4).

Step 2: Truth Table reduction (2^K rows): Truth Table is a data matrix that displays all possible configurations of the condition variables included in the study (6 in our case) represented by 2^K rows, where K represents the number of causal conditions used in the data analysis. There was a total of 2^6 (64) possible combinations of the condition variables for our analysis.

A Truth Table (2^k) was generated, separate for each of the outcome variables - 'Maturity of the SLM planning processes' and 'Data sophistication and use' (Table A3.1). We reduced the Truth Table relating its causal combinations to the intended outcomes at the threshold of 1 as the minimum number of the cases in the specific solution terms to be considered for final analysis, recommended for a frequency of a medium-sized samples (e.g. 10-50 cases, 16 cases in our study)(4). Similarly, the minimum consistency cut-off and the proportional reduction in consistency (PRI consistency) was maintained at >= 0.80 and >= 0.70 respectively(6).

For the outcome 1, when raw - consistency cut-off was set at 0.8, there were 12 combinations with success (outcome =1). However, 2 of the cases (G and L) had the PRI consistency of 0.00. Therefore, we increased the consistency cut-off to 0.85 such that the PRI cut-off maintains at >

0.50 for our data. It gave us a total of 10 combinations for the positive outcome (1) and 6 negative outcomes (0).

In case of the outcome 2, we maintained the cut-off at >= 0.8 and >=0.5 respectively. The latter was maintained at > 0.50 so as to keep three cases (Z, J and K) which has PRI consistency of only 0.58 but are the important cases for our analysis, particularly for the outcome 2, as informed by the in-depth interviews. It gave us 9 success cases for further analysis.

Table A3.1: Truth Table results

Outcome 1: Maturity of the SLM planning processes											
**District size**	**Simplicity of inter-org. environment**	**Alliance Maturity**	**Health of inter-org relationship**	**Fidelity to SLM logic**	**SLM Fit with Planning**	**Out-come**	**Number of Cases**	**cases**	**raw consist.**	**PRI consist.**	**SYM consist**
0	1	1	1	1	0	1	3	Z, J, K	1.00	1.00	1.00
0	1	1	1	1	1	1	3	U, E, D	1.00	1.00	1.00
1	0	0	0	1	0	1	1	B	1.00	1.00	1.00
1	0	1	0	1	0	1	1	P	1.00	1.00	1.00
1	0	1	1	1	1	1	1	R	1.00	1.00	1.00
1	1	1	1	1	1	1	1	Y	1.00	1.00	1.00
1	0	0	0	0	0	0	1	G	0.81	0.00	0.00
1	1	0	0	0	0	0	1	L	0.81	0.00	0.00
0	0	0	0	1	0	0	2	X, Q	0.78	0.40	0.40
0	1	0	0	1	0	0	1	S	0.76	0.00	0.00
0	1	0	0	0	1	0	1	A	0.74	0.00	0.00
**Outcome 2: Data sophistication and use**											
0	1	1	1	1	1	1	3	U, E, D	0.95	0.86	0.86
1	1	1	1	1	1	1	1	Y	0.94	0.76	0.76
1	0	1	1	1	1	1	1	R	0.93	0.80	0.80
1	0	0	0	1	0	1	1	B	0.91	0.76	0.76
0	1	1	1	1	0	1	3	Z, J, K	0.83	0.58	0.58
0	0	0	0	1	0	0	2	X, Q	0.78	0.40	0.40
0	1	0	0	1	0	0	1	S	0.76	0.00	0.00
1	0	1	0	1	0	0	1	P	0.76	0.24	0.24
0	1	0	0	0	1	0	1	A	0.74	0.00	0.00
1	0	0	0	0	0	0	1	G	0.71	0.00	0.00
1	1	0	0	0	0	0	1	L	0.71	0.00	0.00

Step 3: Logical reduction and analysis of configuration

The Truth Tables were then logically reduced using the 'Standard Analysis' procedure (recommended procedure in the fs-QCA package by Ragin (4) that gives three scenarios of the solution configurations viz. parsimonious, intermediate and complex solutions. We selected all the 'prime implicants' combinations and all causal conditions were set to be either 'present' or 'absent' in both models(4). The aim of using QCA in this analysis is not to limit the inference within the parsimonious solution but to untangle the complex pathways (8), therefore we concentrated on the intermediate solution. These results are reported in Tables 1 and 2 in the main text.

We also generated results for the 'absent outcome' (Tables A3.2a and A3.2b below). The analyses followed the same process as above but the different consistency thresholds. For the outcome 1, the consistency threshold was at >= 0.80 and for outcome 2, it was 0.84.

Table A3.2a: 'Absent' Analysis Results of Outcome 1 (Maturity of the SLM planning processes)

Conditions	Solutions		
	1	2	3
DHB Size	●	⊗	⊗
Simplicity of I-O relation	-	-	●
Alliance maturity	⊗	⊗	⊗
Health of I-O relation	⊗	⊗	⊗
Fidelity to SLM logic	⊗	●	⊗
SLM fit	⊗	⊗	●
**DHBs/Cases**	**L, G**	**X, Q, S**	**A**
**Consistency**	**1.00**	**0.88**	**1.00**
**Raw coverage**	**0.53**	**0.57**	**0.45**
**Unique coverage**	**0.20**	**0.16**	**0.08**
**Solution coverage**	**0.83**		
**Solution consistency**	**1.00**		

Table A3.2b: 'Absent' Analysis Results of Outcome 2 (Data sophistication and use)

Conditions		Solutions		
	1	2	3	4
DHB Size	●	⊗	⊗	●
Simplicity of I-O relation	-	●	-	⊗
Alliance maturity	⊗	⊗	⊗	●
Health of I-O relation	⊗	⊗	⊗	⊗
Fidelity to SLM logic	⊗	⊗	●	●
SLM fit	⊗	●	⊗	⊗
**DHBs/Cases**	**L, G**	**A**	**X, Q, S**	**P**
**Consistency**	**1.00**	**1.00**	**0.88**	**0.92**
**Raw coverage**	**0.41**	**0.35**	**0.45**	**0.35**
**Unique coverage**	**0.12**	**0.06**	**0.13**	**0.09**
**Solution coverage**	**0.76**			
**Solution consistency**	**0.89**			

1. Ragin CC. Using qualitative comparative analysis to study causal complexity. Health services research. 1999;34(5 Pt 2):1225-39.
2. Rihoux B, Lobe B. The case for qualitative comparative analysis (QCA): Adding leverage for thick cross-case comparison. The Sage handbook of case-based methods. 2009:222-42.
3. Rihoux B, Ragin CC. Configurational comparative methods: Qualitative comparative analysis (QCA) and related techniques. Vol. 51. Sage Publications; 2008.
4. Ragin CC. User's Guide to Fuzzy-Set/Qualitative Comparative Analysis 3.0. Irvine, California: Department of Sociology, University of California; 2017.
5. Ragin CCS, Davey. Fuzzy-Set/Qualitative Comparative Analysis 3.0. Irvine, California: Department of Sociology, University of California; 2016.
6. Greckhamer T, Furnari S, Fiss PC, Aguilera RV. Studying configurations with qualitative comparative analysis: Best practices in strategy and organization research. Strategic Organization. 2018;16(4):482-95.
7. Wagemann C, Buche J, Siewert MB. QCA and business research: Work in progress or a consolidated agenda? Journal of Business Research. 2016;69(7):2531-40.
8. Rihoux B, Rezsöhazy I, Bol D. Qualitative comparative analysis (QCA) in public policy analysis: an extensive review. German policy studies. 2011;7(3):9-82.
